# Home intervention for children and adolescents with unilateral trans-radial and partial carpal reduction deficiencies

**DOI:** 10.1038/s41598-022-11470-8

**Published:** 2022-05-06

**Authors:** Jessica L. Lukaszek, Jordan A. Borrell, Claudia Cortes, Jorge M. Zuniga

**Affiliations:** 1 School of Pharmacy and Health Professions, Creighton University, Omaha, NE USA; 2 Department of Biomechanics, University of Nebraska at Omaha, Omaha, NE USA; 3 Biomechanics Rehabilitation and Manufacturing Initiative, University of Nebraska at Omaha, Omaha, NE USA

**Keywords:** Health care, Therapeutics

## Abstract

Current training interventions assessing pediatric functional motor skills do not account for children and adolescents with upper limb reductions who utilize a prosthesis. Prosthesis rejection showed that 1 out of 5 prosthesis users will reject their prosthesis due to lack of durability, lack of function, not meeting the participant’s needs, perceived lack of need, and medical restrictions indicating that prosthetic users believed they were more functional without the device. It was hypothesized that an 8-week Home Intervention program will result in significant improvements in gross manual dexterity, bimanual coordination, and the functional activities performed during the program. It was also hypothesized that the novel Prosthesis Measurement of Independent Function (PMIF) score will reflect the Home Intervention performance improvements. Five pediatric participants (ages 5–19 years) with congenital upper limb reductions were fitted with a 3D printed upper extremity prosthesis for their affected limb. Participants then completed the 8-week Home Intervention which included Training activities completed 2×/week for 8 weeks and Non-Training activities completed only at week 1 and week 8. Participant’s times were recorded along with each participant receiving a PMIF score ranging from 0 = unable to complete activity, to 7 = complete independence with activity completion. Results showed a decrease in overall averaged activity times amongst all activities. For all activities performed, individual averaged time decreased with the exception of Ball Play which increased over the 8-week intervention period. There was significant interaction for Home Intervention performance with F = 2.904 (*p* = 0.003). All participants increased their PMIF scores to 7 (complete independence) at the end of the 8 week intervention period. Decreases in time averages and increases in PMIF scores indicate that learning and functional use of the prostheses have occurred amongst the pediatric participants.

## Introduction

The Centers for Disease Control and Prevention (CDC) estimates that about 1500 to 4500 children experience upper-limb reduction deficiency (ULRD) every year in the US and 59% of all limb deficiencies in newborns involve the upper limbs^6,9^. In other parts of the world, such as Australia, Finland, and Canada, reports indicate that 3.4 to 5.3 of 10,000 live-born children suffer upper-limb anomalies^5^. The CDC identified four main challenges experienced by children and adolescents with limb loss: (a) difficulties with normal motor skill development, (b) assistance with daily activities, (c) limitations with certain movements, sports, or activities, and (d) potential emotional and social issues due to physical appearance^6^. Prostheses help children and adolescents with upper-limb reductions to engage in functional activities that are fundamental to normal growth and motor development. Over the years pediatric therapies have moved away from the clinical setting and shifted toward recreating those sessions in the home environment^20^. Home programs and interventions consist of activities that the child completes under the parent’s guidance in the child’s home environment^22^. This allows for a structured practice in the child’s natural environment and has shown to lead to successful learning of intervention activities and meaningful, generalized improvements in function^1,13,21^. However, there is a current gap in research regarding the relationship in children and adolescents between home interventions with prostheses and functionality in activities of daily living. A previous investigation on the adult population completed by investigator Huinink and their team, suggested that when the rehabilitation activities were paired with functional tasks during upper extremity prosthesis training, there was a greater carryover and retention of functional activities even after time periods of non-use^12^. Furthermore, a literature review composed of over 25 years of research in regards to prosthesis rejection showed that 1 out of 5 prosthesis users will reject their prosthesis due to lack of durability, lack of function, not meeting the participant’s needs, perceived lack of need, and medical restrictions indicating that prosthetic users believed they were more functional without the device^3,23^. Functionality with prostheses can grow with the repetition of manual tasks in order to build new neural connections and instill learning as seen in the mechanisms of use-dependent plasticity^24^. Having the ability to introduce the prosthesis and develop functional skills in the child’s comfortable environment can enhance the likelihood of engaging in functional activities that are fundamental to normal growth and motor development^6,7^.

There are several tools currently utilized to assess pediatric motor function and development, prosthesis function, and overall independence with activity of daily living completion. A common evaluation for assessing pediatric functional motor skills is the Goal Oriented Assessment of Lifeskills (GOAL)^19^. This standardized evaluation includes several tasks that the child, aged 7–17 years will encounter in their daily lives. These activities include using utensils to cut, opening key and combination locks, coloring, cutting, folding, and taping a paper project, organizing and filling a three-ring binder, putting on and taking off a shirt and shorts, bouncing and kicking a ball, and carrying a loaded tray while avoiding obstacles. The Peabody Development Motor Scales (Second Edition; PDMS-2) is a standardized assessment used to evaluate motor development and abilities in children from birth to 5 years. It is a revision of the Peabody Developmental Motor Scales (PDMS) originally published in 1983^8^. The assessment is typically used with children with Cerebral Palsy and amongst general Pediatric Rehabilitation. It consists of six subtests which include object manipulation, grasping, and visual-motor integration, from which a Gross Motor Quotient is calculated to measure large muscle systems, Fine Motor Quotient to measure small muscle systems, and Total Motor Quotient of both the Gross Motor Quotient and Fine Motor Quotient to determine overall motor abilities. The child’s scores are compared against normative data related to gender, race, geography, and other variables to determine the child’s strengths and areas for improvement^8^. The Prosthetic Upper Extremity Functional Index (PUFI)^26^ is a questionnaire that was created by researchers to assess how often a child uses their prosthesis in their everyday activities. This included asking parents and children about the completion of everyday bimanual activities with and without the use of their prosthesis along with how useful they perceive the prosthesis to be; this was completed via questionnaire. Participants scoring high on the questionnaire indicate the greatest degree of function with the prosthesis and low scores indicate the least amount of function with the prosthesis^26^. Lastly, the Functional Independence Measure (FIM) has been used to document a measure of a patients’ physical and cognitive function in relation to performance and independence of activities of daily living^14^. Patients can be scored from 1: Total Assistance to 7: Complete Independence, as to how independent they are in the completion of their activities of daily living^14^.

While the tools presented above have been successful in showing their findings, they do contain several limitations when approached by a pediatric participant using an upper extremity prosthesis. These limitations are related to age and subjectivity of children’s abilities leading to a variety of scores based on the assessment evaluator. There is a critical gap in the validity and effectiveness of home interventions for children with upper limb reduction deficiency. Pediatric prostheses users are not taken into account while forming these assessments and because of their limb reduction, may score lower or be placed in a different category than their typical counterparts even if they can function in their everyday activities. A more appropriate intervention is needed to measure and increase everyday functionality of prostheses among pediatric users so that they are not deemed as incapable due to their limb reduction but instead reach a form of normalcy among their typical peers. Thus, the purpose of the current investigation was examine the effectiveness of an 8-week home intervention program for children and adolescents with upper-limb reduction deficiency. The home intervention program was derived using elements from the GOAL and FIM assessments, including other activities that pediatric prosthetic users may encounter in activities of daily living. These activities were timed and scored using the novel Prosthesis Measurement of Independent Function (PMIF) to determine overall ability and functionality.

The purpose of this proof-of-concept study was to test an 8-week home intervention program for children and adolescents with congenital upper limb reductions and to test the newly created, novel PMIF scoring system, which was developed in order to quantify the completion of adapted activities of daily living. It was hypothesized that at the completion of the 8-week Home Intervention program will result in significantly improvements in gross manual dexterity, bimanual coordination, and the functional activities performed during the program. Furthermore, it was also hypothesized that the newly developed PMIF will reflect the Home Intervention performance improvements. Our hypothesis was based on previous investigations showing motor performance improvements using intervention programs to assess pediatric motor function, prosthesis function, and overall independence wen performing activities of daily living^8,14,19,26^.

## Methods

### Experimental design

A group of children and adolescents with unilateral upper-limb reduction performed a unilateral and bimanual coordination task, as well as a gross manual dexterity task with the affected and non-affected sides before and after an 8-week home intervention training. All participants reported non-affected side dominance. This experiment did not use a control group. Pre and post intervention scores were taken. The experimental group performed the home intervention activities wearing their prosthesis on the non-preferred and reduced side.

### Subjects

Five participants with congenital upper extremity reductions (3 boys, 2 girls, 5–19 years of age). Two of the participants had trans-radial reductions and the remaining three had partial carpal reductions (Table 1). All participants had left sided congenital reductions and right sided hand dominance. Hand dominance was reported by the participant at the initial session.

**Table 1:** Congenital upper limb reduction participant demographics and week 1/week 8 total time of activities performed throughout training intervention.

ID	Age (years)	Reduction level	Total time of all tasks completed at week 1 (s)	Total time of all tasks completed at week 8 (s)
**1**	5	Trans-radial	216.5	192.27
**2**	19	Partial-carpal	109.15	105.39
**3**	8	Partial-carpal	171.45	231.71
**4**	12	Trans-radial	133.12	86.41
**5**	17	Partial-carpal	116.92	61.64
		M ± SD	149.43 ± 39.82	135.48 ± 65.19

Inclusion and exclusion criteria followed previous studies involving children with congenital upper limb reductions^30,31^. Inclusion criteria were children and adolescents (male and female; aged 2–19 years) with congenital, unilateral upper-limb reductions of any digit, hand, arm, or shoulder. Any subjects with prior prosthesis experience were included only if they had not used a prosthesis for at least six months prior to conduction of the study. Exclusion criteria included upper extremity injury within past month, medical conditions that are contraindications for wearing a prosthesis (such as skin abrasions and musculoskeletal injuries of the upper limbs), as well as neurological or psychiatric disorders based on parent’s report All children were admitted to the study following informed consent or parental and/or legal guardian written informed consent for both study participation and publication of identifying information/images in an open-access publication, as approved by the Institutional Review Board of the University of Nebraska Medical Center (IRB #614-16-FB). All methods were performed in accordance with the relevant guidelines and regulations. All subjects completed a medical history questionnaire. Parents and children/adolescents were informed about the study and parents signed a parental permission form. For children/adolescents, an assent was explained by the corresponding author and signed by the participant and their parents. Additionally, detailed safety guidelines were given to parents of upper-limb deficient subjects regarding the use and care of the prosthesis.

### 3D printed partial carpal and trans-radial prosthesis

**Figure 1:**
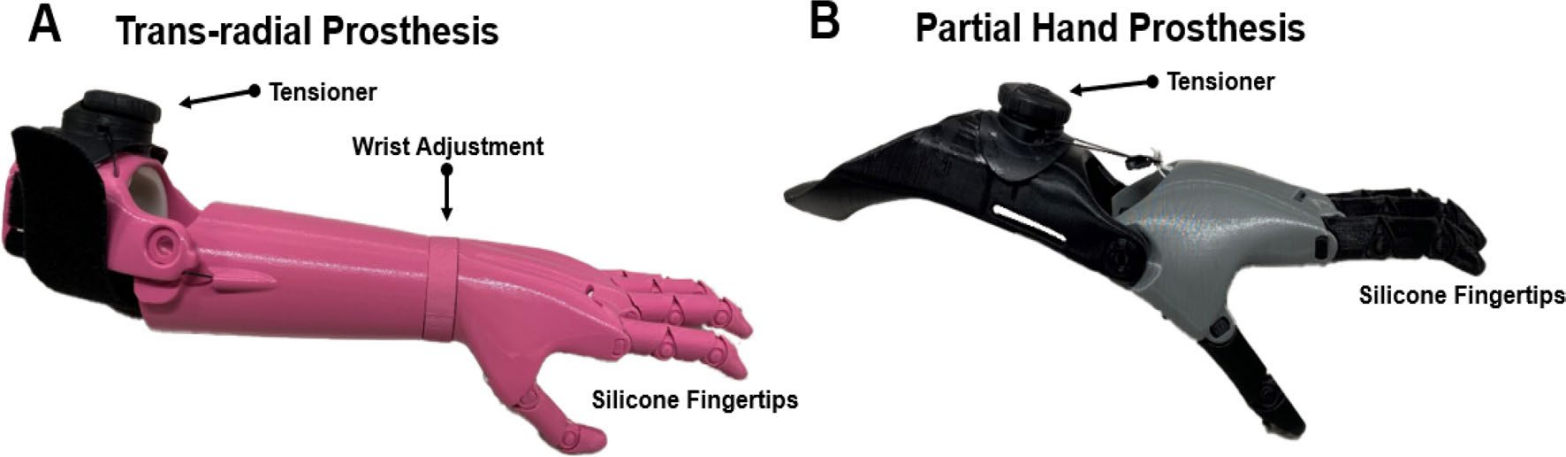
3D printed prosthesis. Exemplar photo of the (**A)** trans-radial prosthesis and (**B)** partial hand/carpal prosthesis used in this proof-of-concept study. Elastic cords placed inside the dorsal aspect of the fingers provide passive finger extension. Finger flexion was driven by non-elastic cords along the palmer surface of each finger and was activated through 20° wrist flexion or elbow flexion of the residual functional joint.

During the first orientation visit, pictures were taken of both the affected and non-affected hands for fitting of the 3D printed hand using techniques from Zuniga et al.^29^. The prosthesis was designed utilizing the measurements of the participant’s affected and non-affected hand. These measurements allowed for an appropriately sized 3D printed prosthetic hand that matched the non-affected hand (Fig. 1). The 3D printed prosthetic hand was designed using a modeling software program (Fusion 360, Autodesk, San Rafael, CA, USA) and manufactured in the researcher’s laboratory using low-cost desktop 3D printers (Ultimaker 2 + Extended, Ultimaker B.V., Geldermalsen, The Netherlands). The material used for printing the 3D hand is polylactide (PLA) plastic. Other components of the prosthetic hand include Chicago screws of various sizes, 1 mm lift nylon cord, 1.5 mm elastic cord, Velcro, medical-grade firm padded foam, protective skin sock, and a dial tensioner system (Mid power reel M3, Boa Technology Inc., Denver, Colorado). The device is body powered via flexion of either the wrist (partial carpal prosthesis) or elbow (trans-radial prosthesis) joints. Elastic cords placed inside the dorsal aspect of the fingers provide passive finger extension. Finger flexion is driven by non-elastic cords along the palmer surface of each finger and is activated through 20–30° of wrist or elbow flexion. The result is a composite fist (flexing the fingers towards the palm) for pinch grasp. Justification for the design and use of the 3D-printed prosthetic hand are low cost, easy usage, easy fitting, easy assembly, and visually appealing to children and adolescents. The files for the design are available online on the National Institutes of Health (NIH) 3D print exchange website^28,30^ and Thingiverse^30^.

### Unimanual and bimanual coordination task

**Figure 2:**
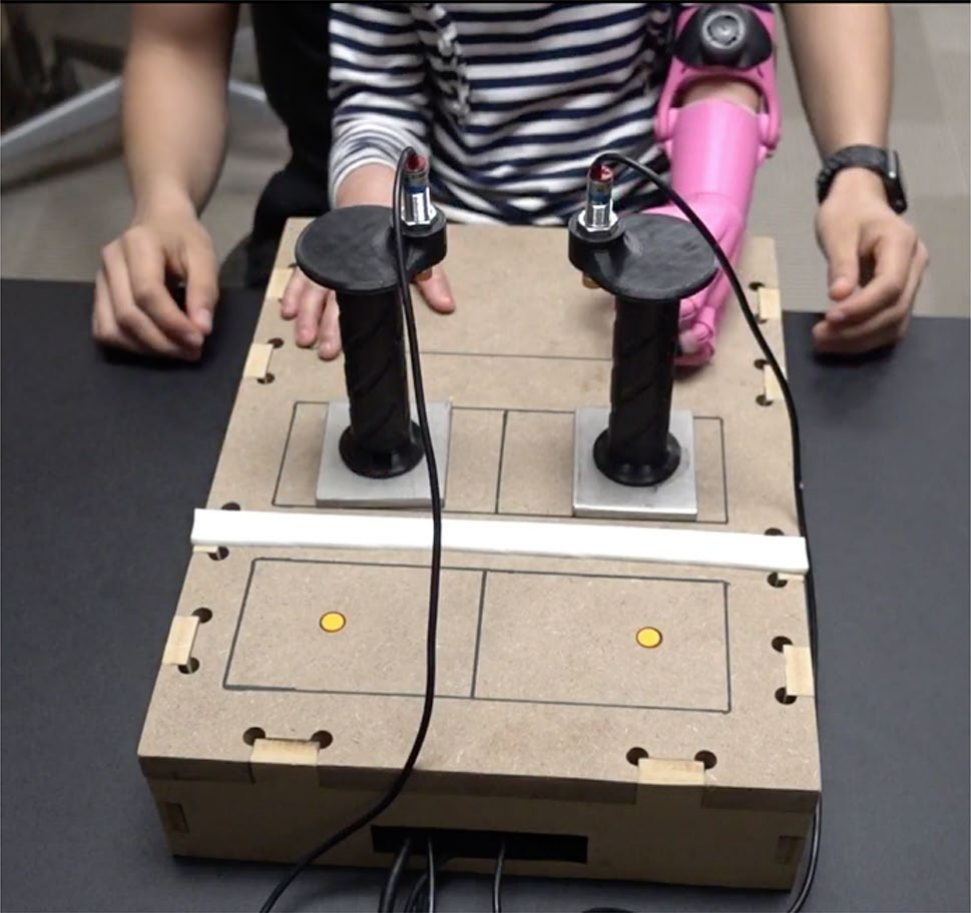
Bimanual reaching task with two small trays. The task involved the participant grasping the handles, transporting the small trays to the top ledge, releasing the tray handles and returning their hands to the standardize start position.

A custom reaching tray apparatus was utilized to assess temporal coordination for both unimanual and bimanual activities^15^. This tray featured two small, steel sheets (10 cm × 5 cm) affixed to the bottom of vertical handles which subjects will use for manipulation. The two steel trays rested on a flat surface composed of medium-density fiberboard. The subjects started at a standardized position and were asked to reach forward, grasp the trays and transport them over a ledge, and finally, return their hands to the starting position. The subjects performed this experiment under three different conditions: unimanually with the non-affected limb, unimanually with the prosthetic limb, and bimanually (Fig. 2). Measurements of inter-limb coordination were adapted from the procedures previously described by Kilbreath and colleagues^15^. Inter-limb coordination was assessed by measuring event trigger timing differences from the position time series data. This was completed at week 1 and week 8 of the Intervention time period.

### Gross manual dexterity task

**Figure 3:**
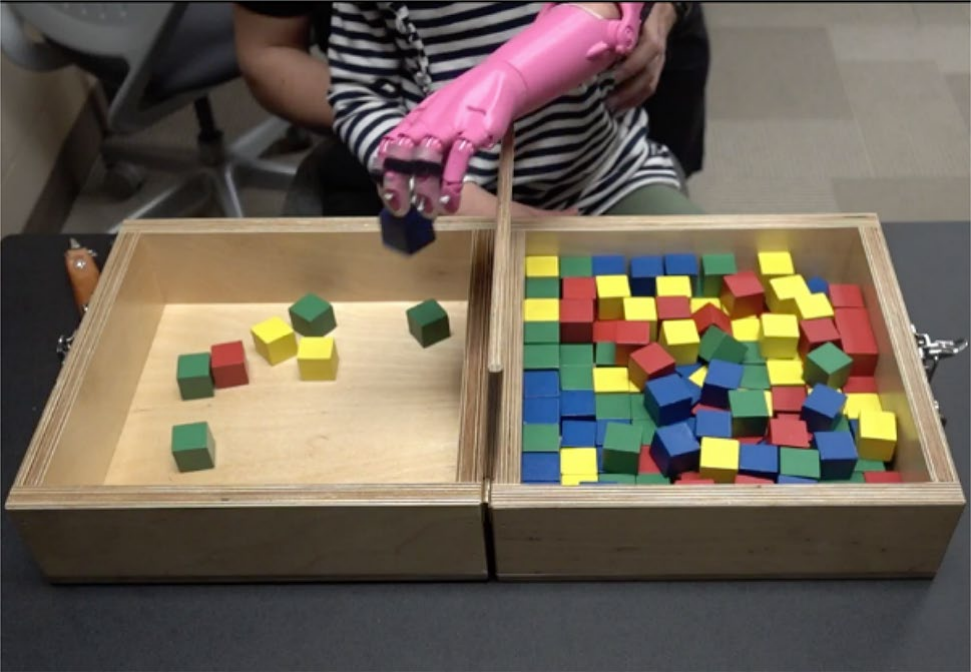
Box and Block test used to test gross manual dexterity being performed by the affected hand. Tasks will involve moving as many blocks as possible from one side of the partition to the other in one minute.

The Box and Block Test has been suggested as a measure of unilateral gross manual dexterity^17,18^ and has been previously used to assess upper-limb prosthetic performance and motor learning^7^. Norms have been collected on adults with neuromuscular involvement and in typically developing children^17,18^. The Box & Block Test consist in a wooden box dimensioned in 53.7 cm × 25.4 cm × 8.5 cm. The partition is placed at the middle of the box, dividing it in two containers of 25.4 cm each. There are 150 wooden cubes of 2.5 cm in size.30 The Box and Block Test provides quantitative data regarding the gross dexterity of the affected and non-affected upper limbs^27,29^. The participant performed the task with the non-affected hand then re-performed the same task with the prosthesis. The B&B task required the subject to move 1-in. blocks one at a time from one box, over a partition, and to drop the blocks in the adjacent box (Fig. 3). The subject was seated comfortably, and then completed a 60-s trial of the B&B task with the unaffected hand followed by the affected hand with the 3D-printed hand. The subject was asked to place their hands on the sides of the box. As testing started with a cue from the researcher, the subject was asked to grasp one block at a time, transport the block over the partition, and release it into the opposite compartment. Protocol followed 3 trials of the Box and Block Task with 60-s of rest between trials for each hand (Fig. 3).

### Home intervention training

The participant was asked to perform modified activities from the Goal-Oriented Assessment of Lifeskills (GOAL) used to assess the performance of instrumental activities of daily living over a period of 8 weeks of prosthetic use (2 times per week)^19^. Participants completed timed activities to assess functional completion of activities of daily living with the prosthesis under the direction of trained study personnel. All activities were timed by telling the participant, “Ready, Set, Go” and ending with “Stop” with the exception of the drawing activity. The participant completed these activities at least twice a week with a team member via webcam to collect the data. Each activity was scored using a novel scale derived from the Functional Independence Measure (FIM)^14^ called the Prosthesis Measurement of Independent Function (PMIF; Table 2). The PMIF scores the participant’s abilities to complete each activity on a range from 7, Independence with the participant utilizing the prosthesis with no assistance, to 1, Complete Assistance with the participant requiring physical touch assistance to utilize their prosthesis and requires total assistance to complete the activity. The activities included Block Building, Utensils, Paper Activities, Ball Play, Tray Carry, and Bike Circuit.

**Table 2:** Prosthesis measurement of independent function (PMIF).

Score	Description
**7**: Independence	Participant utilizes prosthesis with no assistance, safely, and within time expectations
**6**: Moderate independence	Participant completes activity with prosthesis but there are concerns for participant’s safety (using scissors or tape dispenser incorrectly), completed the activity outside of time expectations, or disorganized in movements and completion
**5**: Verbal cueing	Participant requires verbal cueing to complete or stay on course with the activity; Verbal cuing is given but action does not change, if action changes and is completed correctly for the remainder of the activity, score at a 6
**4**: Minimal assistance	Participant requires physical touch assistance to utilize their prosthesis and requires no more than 25% assistance to complete the activity; Hand over Hand assistance to complete approximately a quarter of the activity
**3**: Moderate assistance	Participant requires physical touch assistance to utilize their prosthesis and requires no more than 50% assistance to complete the activity; Hand over Hand assistance to complete approximately half of the activity
**2**: Maximum assistance	Participant requires physical touch assistance to utilize their prosthesis and requires no more than 75% assistance to complete the activity; Hand over Hand assistance to complete more than half of the activity
**1**: Complete assistance	Participant requires physical touch assistance to utilize their prosthesis and requires total assistance to complete the activity; Hand over Hand assistance to complete the entirety of the activity
**0**: Activity did not occur	

### Training tasks

**Figure 4:**
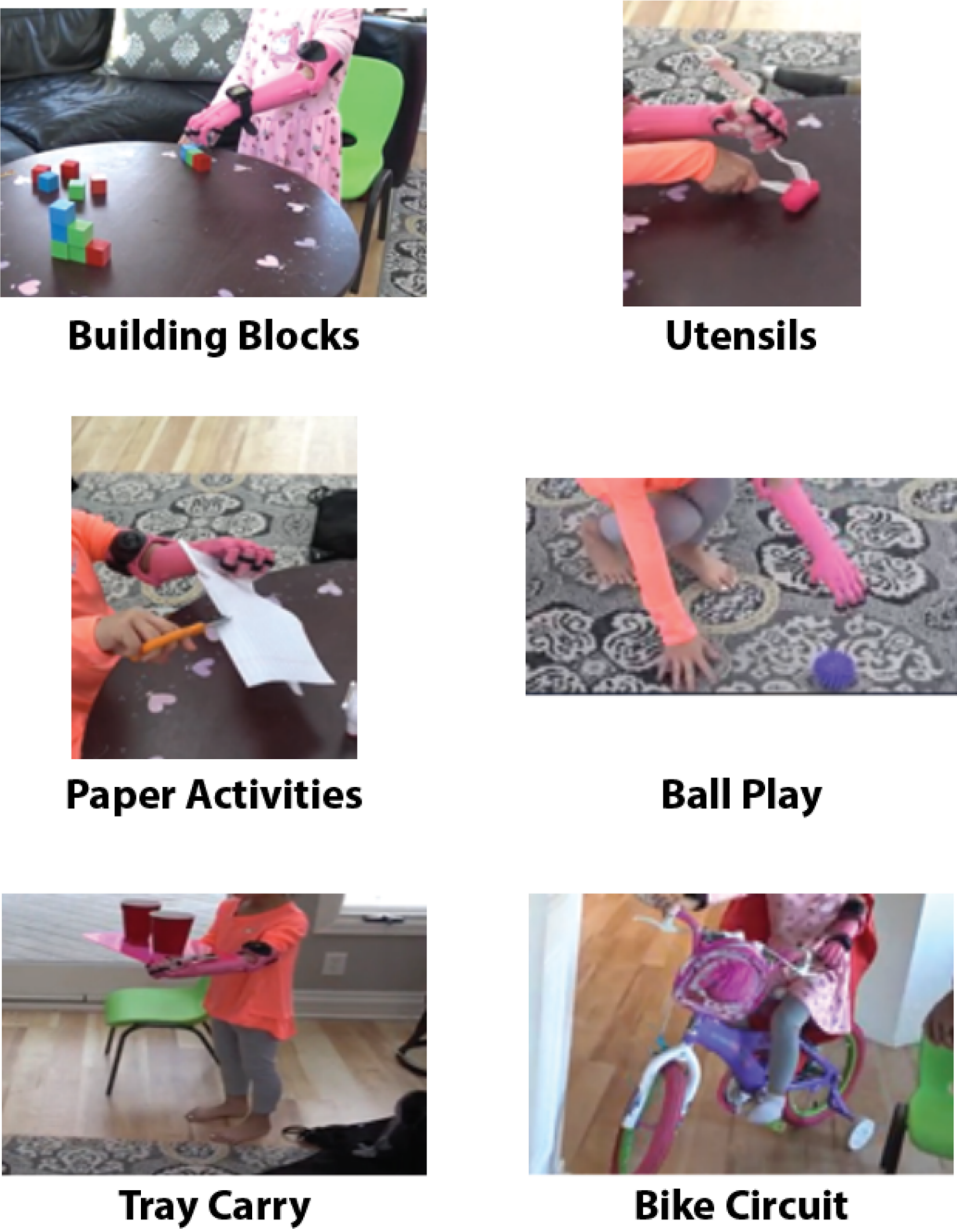
Pictures of the activities completed in the Home Intervention Program including Block Building, Utensils, Paper Activities, Ball Play, Tray Carry, and Bike Circuit.

Utensils consists of using the participant’s unaffected extremity and the prosthetic appendage to make four cuts with a fork and knife while utilizing a universal cuff to grasp the utensil in in the prosthesis (Fig. 4).

Paper Activities consists of several parts, stabilizing a piece of paper while the participant draws on it which was the only untimed activity, folding the piece of paper, stabilizing a tape dispenser with the prosthesis to tear 3 pieces of tape, and holding the paper with the prosthesis while the able hand uses scissors to make 3 cuts (Fig. 4).

In Ball Play the participant must use the prosthesis to pick up a ball from the ground and either underhand or overhand toss it to a partner 3 times in a row (Fig. 4).

Tray Carry has participants walk in a Figure 8 formation around two toys placed approximately 5 feet apart while balancing a tray loaded with two cups between their prostheses and unaffected upper extremity (Fig. 4).

### Non-training tasks

Two intervention activities were only completed at week 1 and week 8 of the Home Intervention to assess the pre and post intervention abilities and time averages of the child’s prosthetic usage.

**Figure 5:**
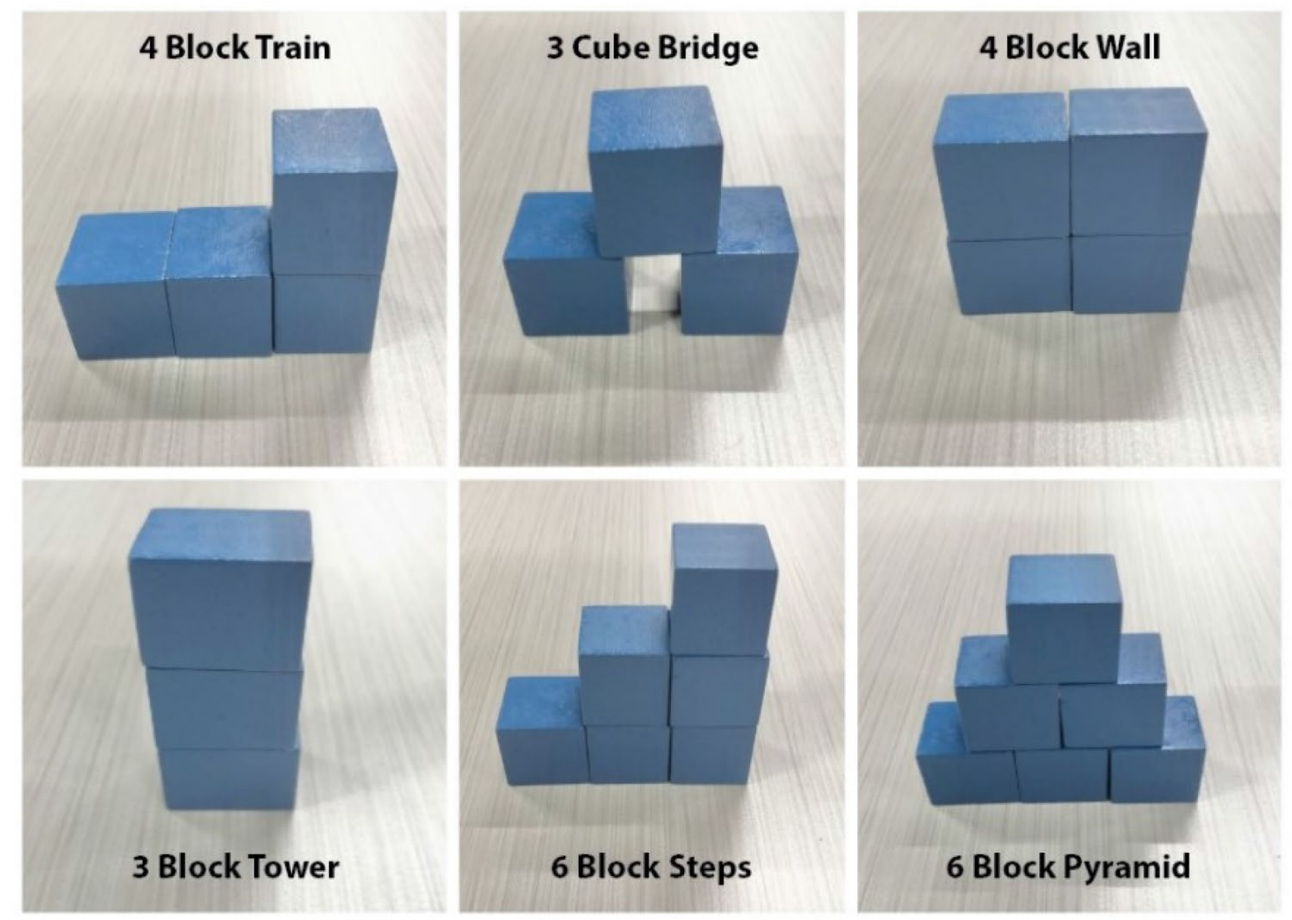
The Block Building activity in which the participant was asked to build a 4 block train (top), 3 cube bridge (middle left), 4 block wall (middle right), 3 cube tower (bottom left), 6 block steps (bottom middle), and 6 block pyramid (bottom right) at both the first and last week of the home intervention period.

The Block Building activities included two trials of a series of 6 different block building activities for each hand separated by 30 s of rest (a total of 18 block building activities per hand). Specifically, the participant was instructed to perform three trials of the following building block activities: 4-block train, 3-cube bridge, 4-block wall, 3-block tower, 6-block steps, and 6-block pyramid (Fig. 5). The Block Building activity was performed during the first and last week of the home intervention.

The Bike Circuit requires participants to either ride a bike or walk a bike in a figure-eight around two stationary objects approximately 5 feet apart to demonstrate independent bilateral forearm activation in order to turn the bike with the able appendage and the prosthesis (Fig. 4). The Bike Circuit was performed during the first and last week of the home intervention.

### Statistical analysis

A Shapiro–Wilk test was performed to analyze all data for normality with a CI of 95%, with the null hypothesis of this test stating that the gross manual dexterity using the limb of interest (non-preferred with device) is normally distributed within the sample population. Similarly, a Levene’s Test of Homogeneity was also conducted to assess variance of the gross manual dexterity using the non-preferred limb before and after the intervention.

A two-way repeated measures ANOVA [2 × 3; Time (pre and post) × Hand (preferred hand, non-preferred, and both)] was used to test time × hand interactions for the coordination task. A separate two-way repeated measures ANOVA [2 × 2; Time (pre and post) × Hand (preferred hand and non-preferred)] was used to test time × hand interactions for the gross hand dexterity task. Post Hoc analysis using a student’s t-test to compare means were conducted on PMIF, gross manual dexterity testing, and non-training tasks. Data were presented as mean ± SD, and an alpha value of 0.05 was considered statistically significant for all comparisons.

### Ethical statement

All children were admitted to the study following informed assents or parental written consent as approved by the Institutional Review Board of the University of Nebraska Medical Center.

### Consent for publication

All contributing authors approve of this publication.

## Results

Demographic characteristics of the sample are outlined in Table 1. All subjects had not used a prosthesis for a minimum of six months before participation in the study.

The Shapiro–Wilk indicated there were no significant results with data from the affected side (*p* > 0.05) for all tasks and trials (pre and post) performed, demonstrating normally distributed data. Normality was similarly observed with data from the non-affected side (*p* > 0.05) for all tasks and trials using the Shapiro–Wilk test. The Levene’s Test of Homogeneity using a 95% confidence interval indicated for all the trials (pre vs post) tested with either hand for each task performed that there were no significant differences (*p* > 0.05) from the null hypothesis. These results suggest that overall conditions were normally distributed and that variances were homogenous.

### Gross manual dexterity and coordination performances

There was not a significant hand × visit interaction for gross manual dexterity performance, F = 0.869, *p* = 0.505. Further analyses revealed that the affected side performance increased, though not significantly, after the 8 week intervention (11.20 ± 4.55 blocks per minute) compared to their baseline performance (8.00 ± 3.67 blocks per minute). There was also not a significant hand × time interaction for bimanual coordination performance, F = 2.024, *p* = 0.229. Further analyses revealed that the reduced side scores slightly lower, but not significant, times after the 8 week intervention compared to the participant’s baseline measurements (Table 3). In agreement with the hypothesis, gross manual dexterity performance and bimanual coordination increased, though not significantly, over the course of the 8-week intervention period.

**Table 3:** Timing of bimanual coordination tasks found from the bimanual reaching task with two small trays.

Subject	Timing of bimanual task (s)			
	Non-affected hand		Affected-hand with prosthesis	
	Before	After	Before	After
1	6.40	4.99	6.82	5.59
2	3.33	2.23	3.21	2.31
3	6.43	5.02	6.8	4.98
4	3.51	3.98	3.62	4.52
5	4.97	3.97	6.08	5.96
Mean	4.93	4.04	5.31	4.67
SD	1.34	1.01	1.57	1.28

### PMIF scores

**Figure 6:**
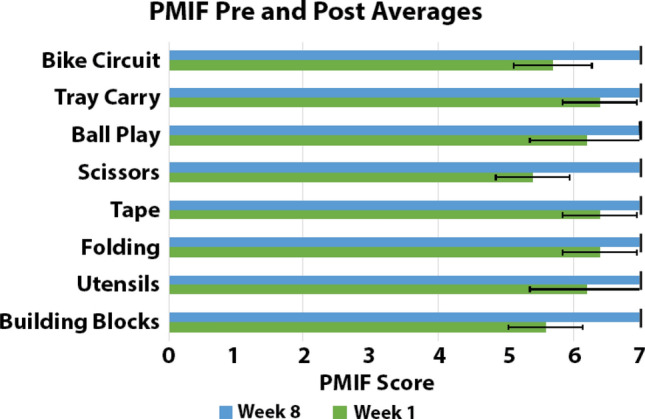
Prosthesis Measurement of Independent Functions (PMIF) pre and post intervention time averages for activities.

The Prosthesis Measurement of Independent Function (PMIF) showed a significant increase from week 1 to week 8 (*p* < 0.001), F = 0.96 across all participant scoring. Participants started with their initial overall PMIF scores at week 1 (average score of 6.04 ± 0.63) and indicated increase of abilities at week 8 overall scoring (average score of 7.00 ± 0.00) (Fig. 6).

### Home intervention performance

There was significant interaction for home intervention performance, F = 2.904 (*p* = 0.003). Post-hoc analysis revealed that participants preformed significantly lower times to complete overall activities at week 8 (135.48 ± 65.19 s) than their initial times to complete overall activities at week 1 (149.43 ± 39.82 s) (Table 4). In agreement with our hypothesis, there was a significant difference (*p* = 0.003) between week 1 and week 8 overall intervention times. When individually analyzed, the two activities of the home program that showed significance between week 1 and 8 were Folding (*p* = 0.046) and Building Blocks (*p* < 0.001) (Table 4).

**Table 4:** Pre and post intervention time averages (mean ± SD) for the 8 intervention activities; * = significance (*p* < 0.05).

Average intervention times (s)		
Activity	Pre (Week 1)	Post (Week 8)
Bike circuit	22.60 ± 2.42	16.45 ± 3.96
Tray carry	9.91 ± 2.54	8.30 ± 3.45
Bally play	15.61 ± 5.80	21.51 ± 13.07
Scissors	6.33 ± 2.29	5.32 ± 1.36
Tape	20.76 ± 13.83	16.04 ± 16.00
Folding	14.11 ± 7.59	4.55 ± 0.87*
Utensils	26.68 ± 14.11	13.42 ± 9.27
Building blocks	33.60 ± 19.23	33.14 ± 20.75*
Overall (M ± SD)	149.43 ± 39.82	135.48 ± 65.19*

### Intervention outcomes

**Figure 7:**
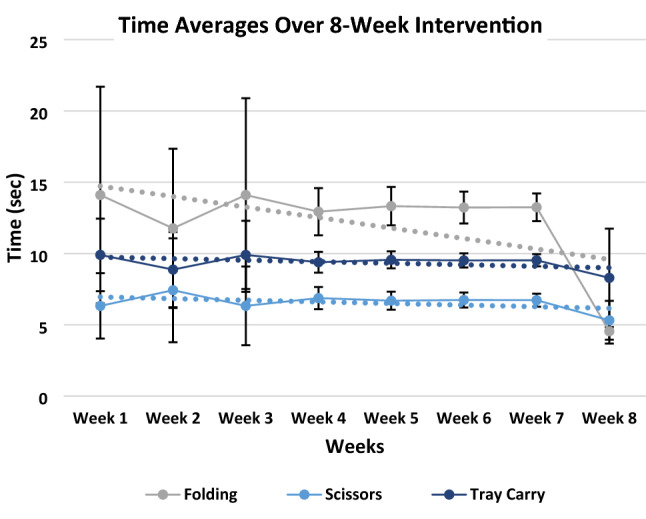
Time averages to complete each activity over the 8-week intervention period. Many time averages for each activity significantly overlapped. Folding, Scissors, and Tray Carry were good representatives of what was typically seen in all activities performed.

The time averages for each individual activity was taken for each of the 8 weeks of the home intervention (Fig. 7). Most home intervention tasks did not have a standardized decrease in time from week to week, but by following the trend lines indicated by the linear fitted dotted lines in Fig. 7, we can see that overall times decreased across Folding, Scissors, and Tray Carry. This decreased trend in time was also seen in Building Blocks, Utensils, Tape, and Bike Circuit with the exception of Ball Play in which the time increased leading to an ascending linear fitted dotted line.

## Discussion

The purpose of this proof-of-concept study was that at the completion of the 8-week Home Intervention program the results indicate significantly improvements in gross manual dexterity, bimanual coordination, and the functional activities performed during the program. The major finding of the present investigation was the significant decrease in time between week 1 and week 8 of the home intervention activities performed with the prostheses (Week 1 = 149.43 ± 39.82 s and Week 8 = 135.48 ± 65.19 s). These significant increases in performance were captured using the novel PMIF (Week 1 = 6.04 ± 0.63 PMIF score and Week 8 = 7.00 ± 0.00 PMIF score). As expected, participants who completed the 8-week intervention showed significant decreases in times for most of the home intervention activities (Table 1). However, the trend of improvements observed in gross manual dexterity and bimanual coordination did not reached significance.

The novel developed PMIF captured the Home Intervention performance improvements which also followed the trend of non-significant improvements in gross manual dexterity and bimanual coordination. The PMIF showed to be a useful rating to quantitatively assess the participant independent function by describing the assistance to complete the activity with the ultimate goal of identifying when the patient utilizes the prosthesis with no assistance, safely, and within time expectations (Table 2).

The current investigation showed significant improvement across all timed home intervention activities except for Ball Play in which the final time ended up increasing compared to baseline (Table 4). The possible reasons for this increase could be due to environmental changes, prosthesis malfunction, participant’s personal attributes, or even an increased focus in completing the activity to their highest potential. Although these methodological concerns can often occur when working with children in their home environment^22^, it can be safely assumed that learning may have occurred with Ball Play even though the time increased. This assumption can be partially verified when analyzing the participant increasing PMIF scores due to the decreased assistance. Participants were receiving more assistance in the beginning of their home program which could indicate why initial time for Ball Play is lower than post intervention time. At week 8 all participants were performing completely independently (PMIF score = 7) on all Home Intervention activities (Fig. 7). This indicates that the increased time in the Ball Play activity could be due to decreased assistance administered to the participant during a relatively complex task. These findings highlight the importance of the novel PMIF scale to complement performance assessments. If the assistance given at week 1 hadn’t occurred, it is assumed that all Intervention Activity times would be greater at week 1 with some participants not even being able to complete some activities due to deficits in their abilities. In this proof-of-concept study, we had a wide age range of participants (5–18 years), and not all of them function similarly due to their differences in developmental milestones and differences in upper limb reductions (Table 1). By utilizing the PMIF it allowed us to individualize the experience for each participant so that they could successfully complete the activities in their home intervention program with hopes of avoiding frustration and burnout. This individualized approach is novel and is not currently implemented in current training interventions^8,14,19,26^.

The purpose of the intervention was achieved as seen by the ultimate decrease in overall activity times from week 1 to week 8 along with an increase in all PMIF scores to complete independence (PMIF = 7 at Week 8). These improvements appeared to translate to all Non-Training activities as well. It has been shown that training can transfer from the trained finger to the non-trained finger^24^. The improved prosthesis performance after training seen in this proof-of-concept study may have similarly transferred from the Training activities to the Non-Training activities.

The intervention itself was inspired by the activities from the Goal Oriented Assessment of Lifeskills (GOAL)^19^. Several activities were eliminated from our intervention due to the inability performing the activity without damaging the prosthesis (opening a 3 ringed binder), inappropriate to fit developmental ages of certain participants (opening key and combination locks), irrelevant to the upper extremities (kicking a ball), or was a skill the participant already possessed and perfected from daily completion prior to receiving their prosthesis (putting on and taking off a shirt)^19^. We also planned for all activities to require bilateral upper extremity activation, with exception of building blocks and box and blocks, in order to complete. This was to encourage the participant to complete the activities as typically as possible and to use the prosthesis in conjunction with their unaffected extremity with hopes of increasing its functionality in the participant’s daily lives.

According to Hadders-Algra, children with unilateral congenital below elbow deficiencies learn to use their affected limb functionally through exploratory activities where they can manipulate objects and use the affected limb as a valuable learning tool^11^. Principles of motor learning suggest that the most functional way to improve the cortical activity and bimanual control is to practice bimanual activities of daily living directly^10^. In this theory, repetition of desired movement and functional tasks are key. These repetitions can be also be justified using the dynamic systems theory indicating, “movement patterns emerge as a result of the interaction between the person’s abilities, the environment and the goal”^16^.

Through Bernstein’s Levels of Construction in motor learning, authors Bongers et al.^4^ found that learning occurs most successfully when there is proprioceptive feedback while using prosthetic limbs so that the brain of the participant can re-learn how to control the prosthetic successfully while completing functional activities. This proprioceptive feedback can be anything from visually watching the action occur with the prostheses to physically moving the prostheses with the able appendage to simulate the desired motion.

Bailes et al.^2^ and Novak et al.^21^ have researched these theories and found that the optimal learning time for interventions to be 1–2 times per week over the course of 8 weeks. This allows the participants to facilitate motor changes in the upper limbs that result in increased coordination and movement functionality^21^. In our research we were successful with this method of having the participants complete the Home Intervention 2 times per week over the 8-week intervention period. It showed to be an appropiate amount of time between sessions where the participant was able to utilize the prosthesis on their own as well as look forward to their individual bi-weekly video meeting with the research personnel.

The information found can help in the development of an assessment derived from Motor and Dynamic Systems theory to better facilitate learning of upper extremity prostheses among pediatric participants in hopes of increasing their functional abilities in their everyday lives.

### Limitations of current study

The limitations of this study was that it was initially planned to be in-person intervention sessions, but due to COVID-19 protocols the intervention was transposed to a virtual platform. This in and of itself brought problems with technology on both ends of the video call. Another limitation was scheduling 2 × weekly intervention sessions with the participants and their caregivers around their personal schedules. This led to many sessions being re-scheduled and even sometimes pushing off the intervention for a few days to accommodate for these scheduling complications. While these were inconveniences to the research, it was necessary for participants to continue participating in life events outside of the research study. At each session participants were encouraged to use their prosthesis outside of the set intervention times as well in their typical daily activities. We received several caregiver reports of the participant’s bringing and using their prosthetics at school, during play activities, and throughout their daily schedules; however, a quantitative assessment for daily use of the prosthesis outside of the home intervention was not measured for this proof-of-concept study.

The participants also appeared to be more comfortable participating in interventions in the comfort of their home environments as compared to a standardized clinical environment. As other researchers have found, participants exhibited successful learning of intervention activities and meaningful, generalized improvements in function when home programs are completed in the participant’s natural environment^1,13,21^.

There was a high degree of variability amongst the participants. This can be lessened in future studies by limiting participants based on age, developmental level, history with prosthetic use, and availability over an 8-week period. This investigation could be improved by including a control group in with the participants. This would allow us to compare typically developed participants to those who utilized the prosthesis to complete similar tasks.

## Conclusion

In conclusion, after an 8-week Home Intervention program, participants showed significant decreases in the overall time for the home intervention activities. Additionally, all participants reached complete independence for all activities on the novel created PMIF score. There was a trend of improvements observed in gross manual dexterity and bimanual coordination, but they did not reach significance. This Home Intervention program allowed for the inclusion of children and adolescents who utilized prosthetic devices and allowed for a form of normalcy among them and their typical peers. This Homer Intervention program adds to the ongoing effort to include children and adolescents who utilize prosthetic devices into rehabilitation programs.

## Data Availability

The raw data supporting the conclusions of this article will be made available by the authors, without undue reservation.
